# Orienting Activity of the Subject as a Mechanism for Instruction, Learning and Development

**DOI:** 10.11621/pir.2022.0403

**Published:** 2022-12-15

**Authors:** Galina V. Burmenskaya

**Affiliations:** a Lomonosov Moscow State University, Moscow, Russia

**Keywords:** Orienting activity of the subject, P.Ya. Galperin’s theory of the stage-by-stage formation of mental actions and concepts, zone of proximal development, types of orientation and types of learning, mental development in ontogenesis, cognitive learning method, Piagetian phenomena, combinatorial thinking, psychological counseling

## Abstract

**Background:**

The 120th anniversary was celebrated in 2022 of the birth of the outstanding Soviet scientist P.Ya. Galperin (1902–1988), who made a significant contribution to the development of Russian psychology.

**Objective:**

To analyze the significance of P.Ya. Galperin’s concept of “orienting activity” for the study of processes of mental development, learning and instruction.

**Design:**

The concept of “the zone of proximal development” (L.S. Vygotsky) is interpreted in light of the doctrine of orienting activity, presenting three examples from different areas of research, where the concept of orienting activity is used to analyze the phenomena of mental development in children and adults.

**Results:**

1. The concept of orienting activity makes it possible to substantially concretize the psychological content and mechanisms of “the zone of proximal development.” 2. The subject’s orienting activity plays a key role, which is implicitly present in the method of “cognitive learning” developed in the Geneva psychological school and reproducing (according to the followers of J. Piaget) “an autonomous process of constructing new operational structures”. 3. The study examines the organization of orienting activity in the process of children’s mastery of the concepts of combinatorial thinking in a learning experiment based on Galperin’s method of stage-by-stage formation of mental actions and concepts. 4. The role of a client’s orienting activity is explicated, and its special organization by the psychologist who is counseling parents on the mental development and upbringing of children and adolescents.

**Conclusion:**

P.Ya. Galperin’s discovery regarding the structure of human activity and introduction of the concept of “orientation,” and the creation of a method for studying the orienting component of action as distinct from the executive component, lead to a much deeper understanding of the central problem posed by L.S. Vygotsky: the interrelation and mechanisms of connection between the processes of learning, instruction (teaching) and development.

## Introduction

The 120th anniversary of the birth of P.Ya. Galperin is a good occasion to look at the scientific heritage of this classic figure in Soviet psychology and to comprehend from a modern standpoint his contribution to science, as well as the potential of his ideas. The publication in the 1960s–1980s of Galperin’s works on the stage-by-stage formation of mental actions and concepts shook the very foundations of Soviet psychology, affecting ideas about its subject, research method, the role of object-oriented actions, the process of internalization, and other basic questions (Galperin, 1968; 1969; 1992; 1998). Galperin’s research opened up a new approach to classical problems and made it possible to see many previously known phenomena in a different, somewhat unexpected light.

For more than half a century, attitudes in Russian science towards Galperin’s ideas have evolved significantly. If at first they were perceived as very controversial and even revolutionary, today they fit naturally into the system of fundamental ideas accepted in Russian psychology (which, however, does not mean they have been deeply understood). Despite the significant difficulties in translating Galperin’s works, there has been interest in them outside the country, including in connection with the ideas of L.S. Vygotsky, whose theory is much better known (Engeness, 2021; Engeness & Lund, 2020; Haenen, 1996; Stetsenko, 2017).

Galperin’s scientific contribution is primarily associated with the theory of planned stage-by-stage formation of mental actions and concepts, as well as the introduction into psychology of the concept of the *orienting activity of the subject*. According to Galperin, orienting activity is an integral and important aspect of any purposeful activity by a subject. He considers orienting activity as the principal *mental function* and the genuine *subject of psychology*, its central category. In order to understand, form, or improve human activity, we must discover the particular characteristics of its orienting basis. Let us briefly recall Galperin’s main theses regarding the concept of orienting activity.

### First

The orienting activity of a subject occurs where and when it is necessary to act in *new* or *changing conditions*; on the other hand, in stable conditions, a learned and automatic action is sufficient, and orientation is not needed.

### Second

In the structure of any human action (more broadly, any human activity that is *not* performed automatically) it is necessary to distinguish between two mandatory components — the orienting and the executive parts of the action.

### Third

The subject’s orienting activity performs several functions: 1) constructing an image of a problem situation or an action being performed; 2) determining significant elements of this field of action from standpoint of the subject’s needs; 3) constructing a plan to solve the problem; 4) monitoring and correcting implementation of the plan. The success of an action in a problem situation depends crucially on the quality of the subject’s orientation — its completeness, generality, and other properties. Being a mandatory (and in this sense universal) mechanism for implementing any action that is *new* for the subject, orienting activity becomes key in situations that require going beyond what has been previously mastered, what is known — i.e., in the course of the subject’s mastering of any new actions, concepts, and competencies. This applies both to attempts at independent learning and the subject’s participation in various forms of purposeful and organized instruction.

Let us consider further, using the example of a number of specific studies, how the application of Galperin’s concept of the subject’s orienting activity leads to a deeper understanding of the classical problem of the interrelation between the processes of instruction, learning, and development.

## Orienting Activity as a Means of Analyzing the Connections among Instruction, Development, and the Zone of Proximal Development

L.S. Vygotsky, the originator of cultural-historical theory, confidently asserted the leading role of instruction in development, but emphasized that this does not apply to all forms of instruction, which can be qualitatively different: one type of instruction (and the learning associated with it) directs processes of development, while another conveys only particular knowledge and narrowly practical skills — that is, it relies on the achievements of development, but does not itself lead to qualitative changes (Vygotsky, 1978, 1984). It is no secret that, for all its great attractiveness and heuristic value, Vygotsky’s position remained largely declarative for a long time. In particular, it lacked a substantive differentiation of the types of instruction that are related in different ways to developmental processes. No hypotheses about possible psychological criteria that distinguish instruction that “promotes” development, from instruction that is not connected with it, appear in Vygotsky’s works. This left Vygotsky’s position abstract and vulnerable to criticism, and most importantly, left open the question of a psychological model of instruction, which is highly sought-after in practice, a model that can not only provide knowledge, but also develop students, prepare the way for ontogenetic changes in their thinking.

P.Ya. Galperin’s introduction of the concept of orienting activity may be viewed as an important step in solving the problem posed by Vygotsky. Galperin showed in numerous works that orienting activity is a defining aspect of the subject’s activity, and that the process of instruction and its effectiveness depend decisively on the content and methods used to orient students, primarily on the completeness of their orientation to the essential conditions of the problem situation or task (Galperin, 1967; 1969). The result of this line of research was Galperin’s concept of *three types of orientation* and the *types of teaching and learning* that correspond to them, which have different developmental effects (1989). Thus, Vygotsky’s thesis about qualitatively *different* types of instruction was fleshed out for the first time.

According to Galperin, traditional instruction is *the first* type of teaching and related learning (Type I), based on an incomplete system of points of orientation and the use of trial and error. Because of this, its success depends on the level of development of the student, rather than affecting that development. The *second* type of teaching and learning (Type II) is based on a full orienting basis of action; the process of mastery proceeds in stages and requires a special organization (by the method of planned and stage-by-stage formation). This type of teaching provides for the formation of full concepts and, although it does not have a strict cause-and-effect relationship with development, nevertheless creates important prerequisites for it. Finally, the *third* type of teaching and learning (Type III) is based on the child’s mastery of a certain *method* of analysis and setting points of orientation in a given subject area. With this type of teaching, the child does not master specific actions or concepts, but *methods of orientation* that enable the student to independently analyze any subject area and solve problems, which ensures advancement in *the development* of thinking (or other processes). Developed by Galperin on the basis of the concept of orienting activity, the conception of three types of teaching and related learning was convincingly demonstrated experimentally and practically by various empirical studies (Obukhova, 2001; Podolskiy, 2012; 2017 et al.).

Similarly, the concept of orienting activity also makes it possible to understand much more clearly the mechanisms of “the zone of proximal development” (ZPD) discovered by L.S. Vygotsky (1984). The idea of the ZPD as a model of the interrelation between instruction and development has been firmly established in psychology, and recognition of the leading role of instruction in the mental development of the child is based on it. However, numerous attempts to further specify its psychological content have not been very successful. We recall that Vygotsky described the ZPD as the discrepancy between the actual and potential levels of a child’s development, as the presence of “immature but maturing processes” (Vygotsky, 1984, p. 267). The difference between a child’s achievements in independent activity and those in cooperation with and under the guidance of an adult points at the ZPD. In connection with the ZPD, Vygotsky emphasized that although each subsequent step in development depends on the level of development already achieved, it is still accomplished through collaboration with an adult.

In such a description, the existence of “the zone of proximal development” is an indisputable fact, but the mechanisms that link processes of instruction and development in it remain undiscovered. As P.Ya. Galperin rightly noted, the existence of a “zone” does not say anything about *how* the child learns and how, as a result, the development of thinking takes place (if it does) (1967). In other words, the main question is *what* a child and an adult do in the course of joint activity, as a result of which “immature processes” are transformed and the child achieves a higher level of development.

This question can be significantly clarified by the distinction between the orienting and executive components of activity proposed by Galperin: it is not the *interaction* of a child with an adult, in itself, that opens up the ZPD for the child, but the *orientation* in their joint activity (for example, a problem to be solved), which the adult transfers to the child in a clear verbal formulation or an example of an action, either purposefully or spontaneously and practically, whether deliberately or unwittingly, de facto. In collaborative activity, it is the adult who directs the child’s actions (leaving aside the question of whether this is done worse or better, effectively or not very well), and the child constructs his “zone of proximal development” to the extent that he grasps points of orientation that are new to him. By directing the child’s actions, the adult essentially *finishes constructing* the child’s incomplete image of the problem situation or required action, identifies significant conditions that the child does not consider (“an immature process”) or, preferably, arms the child with the means to independently identify the points of orientation that he lacked to solve the problem.

Among the points of orientation for the problem situation that the subject of the action must consider must be included its semantic components: *why* and *for what purpose* the action is performed. This is well shown in the study by L.F. Obukhova and I.A. Korepanova (2005), where Vygotsky’s conception of the zone of proximal development is considered from the standpoint of orienting activity. In other words, the orientation necessary to advance the child’s initial (current) level should include not only the subject matter of the tasks to be solved, but also the semantic aspect of the adult’s actions, since in joint activities with the child it is the adult who conveys the meanings and methods of performing the action.

Thus, the transformation of the *orienting* basis of an action also forms a genuine psychological *mechanism of cooperation* — figuratively speaking, its “main nerve” and “point of contact” of the child and the adult as two subjects of the interaction process, whether educational or purely practical (Burmenskaya, 2012, 2019). We stress that explaining the connection between instruction and development in the ZPD on the basis of Galperin’s concept of orienting activity is not a replacement of one metaphor (“maturing processes”) for another (change of the orienting basis of an action). The direct or indirect transfer to a child by an adult of the points of orientation that the child lacks for successful completion of a task is a fully objective process, which changes the effectiveness of learning/teaching activity. It can be organized in different ways (the three types of teaching, according to Galperin), and it can be directed, supervised with respect to content and form. Organization of teaching Type III, the stage-by-stage formation of mental actions and concepts on the basis of complete orienting activity in the subject, may be considered as an optimal model of the process of interaction between child and adult taking place in the ZPD, and at the same time as instruction leading to development, in Vygotsky’s words.

## Analysis of the Method of “Cognitive Learning” in Light of P.Ya. Galperin’s Theory of Stage-by-Stage Formation of Mental Actions and Concepts

Jean Piaget — the creator of the operational theory of intellectual development in ontogeny and the famous opponent of L.S. Vygotsky — argued for many years that “learning is subordinated to development, and not vice versa” (Piaget, 1972; 1974). There is no doubt, however, that this position on the issue of the relationship between mental development and instruction was largely influenced by the fact that Piaget considered only traditional forms of instruction. With regard to the latter, Piaget quite reasonably denied the possibility of influencing the development of the operational structures of the intellect. It is indicative, however, that over time, the study of mechanisms of intellectual development led representatives of the Geneva school to attempt to create their own version of instruction related to intellectual development. The method, called “cognitive learning” by B. Inhelder and other collaborators of Piaget, creates, in their view, the conditions in which the intellectual structures existing in children are transformed into structures of a higher order (Inhelder et al., 1974). So what are the mechanisms by which the method of cognitive learning produces new operational structures?

To answer this question, we performed a special series of experiments (Burmenskaya & Kurbatova, 1983) to reproduce the procedure for cognitive learning by pre-schoolers of the concept of conservation of quantity, which is described in detail by B. Inhelder and her colleagues (Inhelder et al., 1974). The picture and the results of cognitive learning almost completely coincided with the results of experiments by the Geneva authors, but at the same time clearly showed that they can be described and interpreted quite differently if we approach the analysis of cognitive learning by taking into account the role that orienting activity plays in it.

Let us consider the fundamental thesis of the Genevan method of cognitive learning, that children have to *independently* discover the conservation of quantity. To achieve such independence, according to those authors, there must be a problematic presentation of the material that provokes a cognitive conflict with respect to the task and the child’s attempts to coordinate different ways of reasoning (schemas of action). Along with the experience of solving problems, all this leads children to the gradual development of new, more advanced structures of thinking.

However, the picture of the process of cognitive learning that we reproduced by us, and even the protocols of the experiments of the Geneva authors themselves, showed that in reality the children were not completely independent (Inhelder et al., 1974). The experimenter interacted with the children and, to use Galperin’s terms, actually tried to expand their orientation to the material of the task, to draw their attention to those aspects of the task that they were not taking into account. In other words, the authors of the cognitive learning concept wanted the child to more fully consider the essential features (points of orientation) of the task. This was achieved, for example, by skillful selection of material, as free as possible from the variety of properties of natural objects, in order to make it easier for children to isolate the essential parameters. Another way of directing the child to the desired parameter of an object was quite traditional: while solving the problem, the experimenter asked the child questions that directed their attention to those elements or relationships that were significant for solving the problem.

Thus, the role of orienting activity was obvious and crucial, but the authors of cognitive learning theory did not differentiate it from the general activity of the child and did not recognize it as one of the main conditions for cognitive progress, due to the lack of a distinction between the orienting and executive components of activity in Piaget’s theory.

Nevertheless, comparison of the theories of Galperin and Piaget (in this context) shows their potential complementarity. Despite fundamental differences, these very different approaches to understanding developmental instruction and learning process have in common object-oriented actions, recognition of the significance of cognitive conflict, rejection of the communication of knowledge in a finished form and of the direct guidance of students’ actions. At the same time, Piaget’s theory is insensitive to the obvious manifestations of children’s living, orienting activity, which plays a decisive role in their intellectual progress. However, research shows that it is the concept of orienting activity that allows us to understand more deeply the real psychological mechanisms of “cognitive learning” and, figuratively speaking, to look inside the developmental process, which Piaget considered “an autonomous process of constructing new operational structures,” but which in reality is based on orienting activity and requires the skillful organizing participation of an adult.

## Orienting Activity in the Acquisition of New Concepts: An Example of Formation of the Foundations of Combinatorial Thinking

The formation of combinatorial concepts in children of primary school age and adolescence (Burmenskaya & Evdokimova, 2007) can serve as an example of a specific application of Galperin’s theory to the study of mental development. It is well known that children do not master the elements of combinatorial thinking before the stage of formal operations in adolescence (Inhelder, 1976; Inhelder & Piaget, 1958). At the same time, the teaching of combinatorial mathematics in secondary school encounters very significant difficulties, so that the topic of combinatorial mathematics is often excluded from the compulsory mathematics curriculum in secondary school. Unsuccessful attempts by the majority of schoolchildren to master the basic combinatorial configurations (placement, permutation, combination) are usually attributed to the “unpreparedness” of children’s thinking to master these difficult concepts.

Our analysis of the problem from the standpoint of Galperin’s theory of stage-by-stage formation has shown that children’s difficulties are largely due to their insufficient initial *orientation* to the material of combinatorial problems (Burmenskaya & Evdokimova, 2007). To master combinatorial concepts, pupils must confidently identify the basic properties in sets — volume (size), composition, order and repetition of elements in the set — and also clearly relate the set to its parts (groups or subsets). But in the process of their independent spontaneous and practical mastery of the objective world, children usually do not gain sufficient experience in compiling sets, and are therefore poorly oriented to their properties. Methods of school instruction also do not assume any special familiarization of children with the properties of sets; instead, combinatorial concepts are explained in mathematics curricula using the example of individual tasks and are often introduced immediately in the form of tables, diagrams, or even formulas.

**Figure 1.**
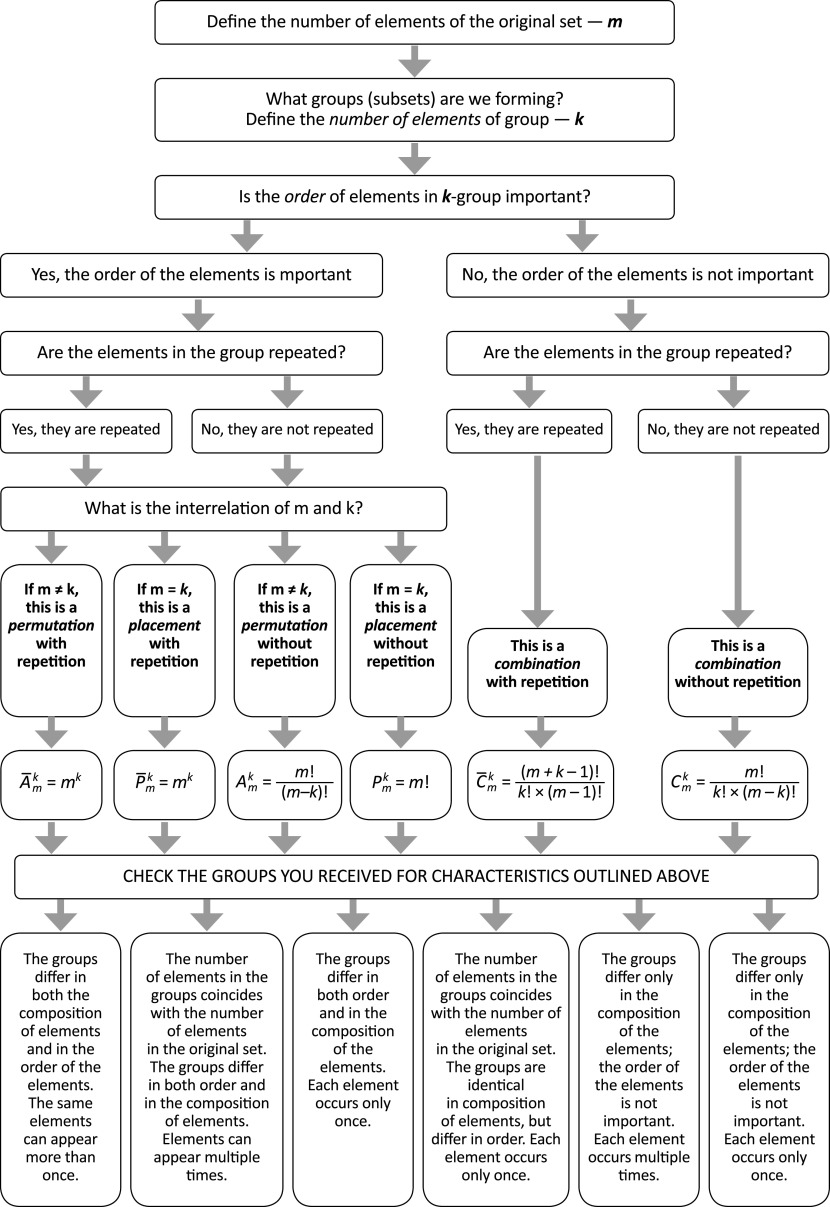
Schema of a complete orienting basis of action of combinatorial configuration analysis (the formulas were introduced only in the version of the methodology for adolescents)

The necessary orientation to combinatorial material can be successfully shaped by Galperin’s method. The basis for construction of such an orientation is the children’s mastery of a special *action to compile a variety of sets* from different elements. This refers to the three main types of combinatorial configurations — placements, combinations, and permutations. At first, the children put them together from various material objects and elements, *focusing on differences in the configurations*, and then they write them down using the appropriate notation. Thus, development of the logical prerequisites of combinatorial thinking begins with special *propaedeutics* (before the introduction of tables and formulas)*,* during which the children themselves make up sets of different types, acquiring real, practical experience of operating with sets. Only after this is the schema of the complete orienting basis of action for compiling combinatorial configurations introduced, in which four kinds of conditions are taken into account: 1) the properties of the initial *set* of elements, its qualitative composition and number of elements; 2) the properties of the formed *groups* (subsets): their qualitative composition and the number of elements included in them; 3) the possibility of *repeating elements* in the compiled sets; and 4) the significance of the *order of elements* in the groups (Fig. 1).

Thus, in contrast to traditional teaching, where it is erroneously assumed that children independently become sufficiently oriented to the properties of sets to start the instruction process, in the experimental set-up following Galperin’s method, that orientation was specially organized by preliminary propaedeutics. It is important that the orientation to combinatorial concepts was then graphically presented in a single schema with a clear indication of similar and distinctive features of all three types of combinatorial configurations. The children’s assimilation of them occurred in the process of solving problems of various types based on the complete schema for the orienting basis, without first memorizing it (the second type of teaching). Using this schema of orienting activity, the students were able to independently analyze and solve combinatorial problems, thereby learning *a generalized* method for solving combinatorial tasks of various types. This method was successfully applied in the instruction of 4th- and 8th-grade students at a general school, with the difference that adolescents’ mathematical training allowed them to use the combinatorial formulas. As a result, the stage-by-stage formation of the action of compiling groups from sets (together with the previous propaedeutics) ensured the students’ full assimilation of the concepts of mathematical combination, which, as was shown, for example, in the works of B. Inhelder (1976), are very complex and often simply inaccessible by other methods.

A delayed test (three months later) of the quality of the learned combinatorial concepts confirmed their strength and their generalized character. Moreover, there was a tangible positive effect of the children’s participation in the formative experiment solving J. Piaget’s tasks and J. Raven’s test. What could explain the children’s cognitive progress? We believe that the generalized character of the methods of orientation learned by the children allowed them to freely transfer to new material the ability to clearly separate out and combine its characteristics — that is, to take into account several features at the same time. During the formative experiment, the children mastered *orientation in a system of signs*, which made an area of combinatorial mathematics that was new and difficult for them accessible, not only in adolescence, but also at primary school age.

## Organization of the Orientation Process as a Condition for Effective Psychological Counseling

Since any new action or an action in changed conditions requires active orienting activity from the subject, its role should be analyzed not only in the traditional context associated with instruction, with mastering new mental actions and concepts, but, in fact, wherever the subject confronts *a lack of his accustomed methods* of response and the need to go beyond them (Galperin, 1998). We suggest that this is also true of those complex processes that occur during the interaction of a psychologist and a client during psychological counseling. We consider this using the example of counseling parents about problems in the development and upbringing of children and adolescents (Burmenskaya, 2017).

Parents usually resort to psychological counseling when they have exhausted their own abilities to solve a problem — for example, to improve their relationship with the child, to understand the reasons for the teenager’s undesirable behavior, or to eliminate family conflicts. Regardless of the significant variety of children’s problems and parental difficulties, psychological help is almost always associated with the need to change the notions that parents have when they come to a psychologist. These notions may involve the essence and causes of the current situation, the parents’ own role in it, or those characteristics of the child that, for example, prevent him or her from being successful at school, having harmonious relationships with relatives or peers, etc. The common task for all these cases is to help the parents to better understand the problematic situation, to understand more deeply the motivations and meaning of the child’s actions and emotional experiences, the nature of the relationship, etc. In a word, the parents need a different, more complete and correct image of the problem situation as an *orienting* basis for its appropriate transformation, for change. In Galperin’s terms, what is necessary is a *reorientation* of the parent*,* which the clinical psychologist should encourage.

In clinical practice, it is well known that this problem cannot be solved by simply *providing information* to the parents: all the correct explanations and recommendations that are offered to them in “ready-made” form are most often understood formally, as external, and remain alien until they become the result of the parents’ own analysis and comprehension, the product of their own activities. But how can that be organized? How can a clinical psychologist help a parent to better understand the problem situation and how to resolve it, while avoiding two unacceptable extremes: attempting to directly transfer knowledge in a finished form (advice) and relying on a parent’s spontaneous insight that has no foundation?

This problem can be solved by using a special form of organization of clients’ activity in the process of interaction with the psychologist. Of course, we are not talking about a literal reproduction of Galperin’s method of stage-by-stage formation for guidance of client-parents’ activity, but about implicit methods *to direct their orientation* to significant circumstances or conditions, to expand the orienting basis of their behavior in the problem situation.

To achieve this, the psychological content of the consultative process is constructed so as to single out and *objectify* for the parents the significance of circumstances or the child’s psychological characteristics that the parents are not sufficiently considering (the missing points of orientation). The consultative interaction takes the form of a special *collaborative discussion and analysis of the problem situation* by the psychologist and parents. The subject for discussion is specially selected fragments of materials for a comprehensive diagnosis of the child and parents, including the results of studies of child-parent relationships and other data. The materials (results of questionnaires, individual statements and reactions of the child, the child’s drawings, emotional and behavioral manifestations, etc.) are presented not in oral retelling by the psychologist, but in a graphic, text or audio format, presenting to parents a kind of materialized projection of the child’s inner world. The parents are invited not only to familiarize themselves with these materials, but also to comment on them, to give their own interpretation, to express their understanding, emotional attitude, etc. Active conceptualization of a problem situation in dialogue with the counselor helps parents to see the situation from a new angle, including looking at themselves and their style of upbringing “through the eyes of the child.” In other words, working through diagnostic materials together helps parents to see essential aspects of the problem situation that had not previously been clear to them, and contributes to the formation of a more complete and accurate orientation to the nature and causes of the problem.

This practice, of course, has its limitations: the success of counseling remains dependent on the difficulty of the problem, the motivation and sensitivity of the parents, their ability to reflect, and many other things. It is beyond dispute, however, that the psychologist’s purposeful consideration *of orienting processes implicitly occurring* during counseling, and conscious efforts to *create conditions for a more complete and appropriate orientation* by parents in a problem situation, almost always contribute to a successful outcome.

## Conclusion

P.Ya. Galperin is credited with discovering the subject’s orienting activity as a principal function of the psyche and a subject of psychology. Today, this concept is part of the conceptual core of Russian psychology and has been recognized as a fundamental category, without which modern ideas about the nature and development of the psyche are inconceivable (Podolskiy, 2017). The studies presented in this article show that the orienting activity of the subject can also be considered as *a mechanism* of processes of the teaching/learning activity, and development. Orientation necessarily participates in the development of any actions, concepts or competencies that are *new* to the subject, and it gradually prepares the way for ontogenetic changes. Orientation processes take place during the child’s cooperation with an adult (“the zone of proximal development”); they actively unfold in the consultative interaction of the psychologist and the parents; they are present in the method of “cognitive learning” and are given an extremely explicit and clear form in specially organized planned stage-by-stage formations (for example, the formation of combinatorial concepts). Orientation processes are not just connected to instruction in the broadest sense of that word, but play a leading role in it.

Galperin was critical of traditional observational psychological research, because he believed that attempts to study *already-formed* and largely automatic processes were limited in principle (1998). He ironically called such attempts “peeking” and, as an alternative, worked out the method of planned stage-by-stage *formation* of mental actions and concepts, which was the basis for the main results of his research. Thus, the concept of the subject’s orienting activity was firmly connected with the formative type of experiment, the only method that makes it possible to openly trace the subject’s orienting activity in the process of formation of new mental actions and concepts.

However, given the absolute dominance in our time of observational research and the laboriousness of formative and instructional experiments, we would like to emphasize that even *beyond* a strategy of formation, the concept of *orienting activity* retains its constructive value. Experience in applying Galperin’s theory demonstrates the productivity of analyzing any human activity for the content of its orienting basis, as well as the effectiveness of not only direct, but also indirect *organization* of the subject’s orientation.

Galperin’s methodology, stressing the crucial significance of organizing the orienting aspect of the subject’s activity, works successfully in scientific research, education, and many areas of applied practice. However, the potential of this approach is far from being fully realized. To move in this direction, analysis of the content of the subject’s orientation and the possibilities of its optimal organization should become a priority of researchers and practitioners.
